# CBFS: High Performance Feature Selection Algorithm Based on Feature Clearness

**DOI:** 10.1371/journal.pone.0040419

**Published:** 2012-07-06

**Authors:** Minseok Seo, Sejong Oh

**Affiliations:** Department of Nanobiomedical Science and WCU Research Center of Nanobiomedical Science, Dankook University, Cheonan, South Korea; University of Westminster, United Kingdom

## Abstract

**Background:**

The goal of feature selection is to select useful features and simultaneously exclude garbage features from a given dataset for classification purposes. This is expected to bring reduction of processing time and improvement of classification accuracy.

**Methodology:**

In this study, we devised a new feature selection algorithm (CBFS) based on clearness of features. Feature clearness expresses separability among classes in a feature. Highly clear features contribute towards obtaining high classification accuracy. CScore is a measure to score clearness of each feature and is based on clustered samples to centroid of classes in a feature. We also suggest combining CBFS and other algorithms to improve classification accuracy.

**Conclusions/Significance:**

From the experiment we confirm that CBFS is more excellent than up-to-date feature selection algorithms including FeaLect. CBFS can be applied to microarray gene selection, text categorization, and image classification.

## Introduction

The fundamental goal of feature selection is to select useful features and eliminate useless ones in a high-dimensional dataset to improve the performance of learning models by alleviating the effects of dimensionality, enhancing generalization capability, speeding up the learning process and improving model interpretability. Typical application areas of feature selection are gene selection from microarray data and text categorization.

In machine learning literature there are three general approaches to feature selection: filters, wrappers, and embedded methods [1], [2]. *Filter methods* select the optimal feature subset based solely on the dataset by evaluating each future based on specific statistics, but completely independently from the classification algorithm. In contrast, *wrapper methods* make use of the algorithm that will be used to build the final classifier to select a feature subset. When compared to filters, they tend to be more computationally expensive, but provide superior performance [3] since they are injected inside the learning algorithm and well suited to the interest of the classifier. In the *embedded technique*, the search for an optimal subset of features is built into the classifier construction, and can be seen as a search in the combined space of feature subsets and hypotheses. Just like wrapper approaches, embedded approaches are thus specific to a given learning algorithm.

Filter Methods are mostly a popular approach because they are simple and fast to extract target features. FSDD [4], Relief [5], and MRMR [6] are up-to-date feature selection algorithms that belong to the filter methods. FSDD is a distance discriminant method. This algorithm calculates the grade of each feature using a distance matrix. The criterion used for selecting good features is *d_b_ – ßd_w_*, where *d_b_* is the distance between different classes, *d_w_* is distance within classes, and *ß* is a user defined value that is usually set to 2 and used to control the impact of *d_w_*. The higher the value of *ß*, the more the focus should be on the distance within classes. The Relief algorithm recursively and randomly selects an instance and identifies its nearest neighbors, one from its own class and others from different classes. The quality estimator in this algorithm is then updated for all attributes to assess how well the feature distinguishes the instance from its closest neighbors. In each iteration, an instance *x* is selected randomly, and its nearest instance is found from the same class (NH), as well as different classes (NM). Finally, the weight value is updated by the equation. Because of this recursive characteristic, runtime is slow compared with the other feature selection methods. Also, results are different every time because the method randomly selects features. MRMR [6] has been proposed to solve the problem by (using mutual information) maximizing the mutual Euclidean distance and minimizing the pair-wise correlations of the features. The minimum redundancy condition is
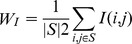
, where *S* denotes the feature subset, and *I(i, j)* is the mutual information of two variables *i* and *j*. To maximize the total relevance 
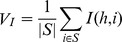
, where *I(h, i)* represents the mutual information between targeted classes *h* and gene expressions *i.*

FeaLect [7] is a very high quality wrapper method. FeaLect proposes an alternative algorithm for feature selection based on the Lasso [8] for building a robust predictor. Lasso is an L1-regularization technique for linear regression which has attracted much attention in machine learning and statistics. Although efficient algorithms exist for recovering the whole regularization path for the Lasso, finding a subset of highly relevant features that lead to a robust predictor is an important aspect to investigate. The hypothesis of FeaLect is that defining a scoring scheme that measures the quality of each feature can provide a more robust selection of features. The FeaLect approach is to generate several samples from the training data, determine the best relevance-ordering of the features for each sample, and finally combine these relevance-orderings to select highly relevant features.

**Figure 1:**
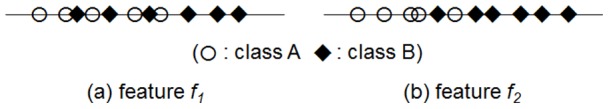
Clearness of feature *f*1and *f*2. Mixed area of *f*1 is larger than *f*2, and it means feature *f_2_* is clearer than *f_1_*.

In this paper, we propose a clearness-based feature selection (CBFS) algorithm which can be classified as a filter method. In our context, clearness means the separability between classes in a feature. If (clearness of feature *f*_2_) > (clearness of feature *f*_1_), then *f*_2_ is more advantageous to classification than *f*_1_. In Fig. 1, feature *f_2_* is clearer than *f_1_*. **Ο** and ⧫ are data samples in *f*_1_ and *f*_2_, and mixed area of *f*_1_ is larger than *f*_2_. Therefore, the classification accuracy using *f_1_* may be lower than *f_2_*. In the CBFS method, we measure clearness of each feature in a dataset, and select top ranked features. CBFS calculates the distance between the target sample and centroid of each class, and then compares the class of the nearest centroid with the class of the target sample. The matching ratio of all samples in a feature becomes a clearness value for the feature. We describe the detailed process to obtain clearness values in materials and methods section.

The proposed method can be used to combine other feature ranking algorithms. We combine proposed methods with *R-value* [9] and validate the improvement of classification accuracy. *R-value* is one of the feature ranking algorithms and it also measures the clearness of each feature by different way of CBFS. It considers nearest neighbor samples of target sample to decide whether it is located in congestion area or not. In some cases, *R-value* based feature selection produces better accuracy than CBFS, and we can expect that combining them improves classification accuracy.

## Materials and Methods

### Clearness-based feature scoring scheme

**Figure 2:**
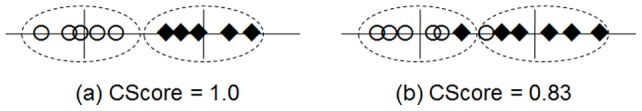
CScores of two features. Fig. 2(b) has two mis-clustered samples whereas Fig. 2(a) has clearly clustered samples.

As mentioned, the proposed method can be classified as a filter method. Every filter method has a scoring scheme for each feature in a dataset. CBFS adopts CScore. CScore(*f_i_*) is a scoring function for feature *f_i_* which measures clearness of the feature. The intuitive meaning of CScore for feature *f_i_* is the degree of correctly clustered samples to the centroid of their class in *f_i_*. In the context of CBFS, each sample is clustered to the nearest centroid of the class. If a sample of class A is clustered to the centroid of class B, it is a mis-clustered sample. In Fig. 2(b), two samples are mis-clustered whereas all samples are correctly clustered in Fig. 2(a). It is clear that well clustered features bring high classification accuracy.

Let's suppose a dataset *DS* has *n* samples, *m* features, and *p* classes. *DS* can be denoted by a set of sample ***x*** *_i_*.

*DS*  =  {***x*** *_1_*, ***x*** *_2_*, .., ***x*** *_i_* ,.. , ***x*** *_n_*}

Each sample is a vector value which has *m* elements (features).

***x*** *_i_*  =  (*x_i1_*, *x_i2_*, .., *x_ij_* , .. , *x_im_*)

A set *CS* contains class labels corresponding to samples in *DS*.

*CS*  =  {*c_1_*, *c_2_*, .., *c_i_* ,.. , *c_n_*}

A class label is a sequential numerical value and the range is [0, *p*-1]. Now we introduce the procedure to obtain CScore(*f_i_*).

#### Step 1

Calculate centroid of each class. It is the same as the median point of a class and calculated by the average operation. Med(*f_i_*, *j*) denotes the median point of class *j* in the feature *f_i,_* which is calculated by:
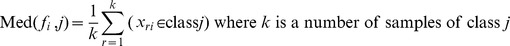
(1)


#### Step 2

Calculate the predicted class label for each *x_ij_* in sample ***x*** *_i_*. After calculating the distance between *x_ij_* and Med(*f_j_*,*c_i_*) for all classes, we take the nearest centroid Med(*f_j_*, *s*) and *s* is a predicted class label for *x_ij_*. The distance between *x_ij_* and Med(*f_j_*, *t*) is calculated by:
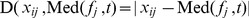
(2)


As a result of step2, we have *n* × *m* matrix M_1_ and element value M_1_(*i*,*j*) is predicted class label for *x_ij_*.

#### Step 3

Calculate *n* × *m* matrix M_2_ which contains a matching result of predicted class label and correct class label in *CS*. M_2_(*i*,*j*) is calculated by:
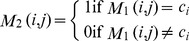
(3)


#### Step 4

Calculate CScore(*f_i_*). Finally we calculate CScore(*f_i_*) by:
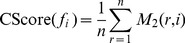
(4)


**Figure 3:**
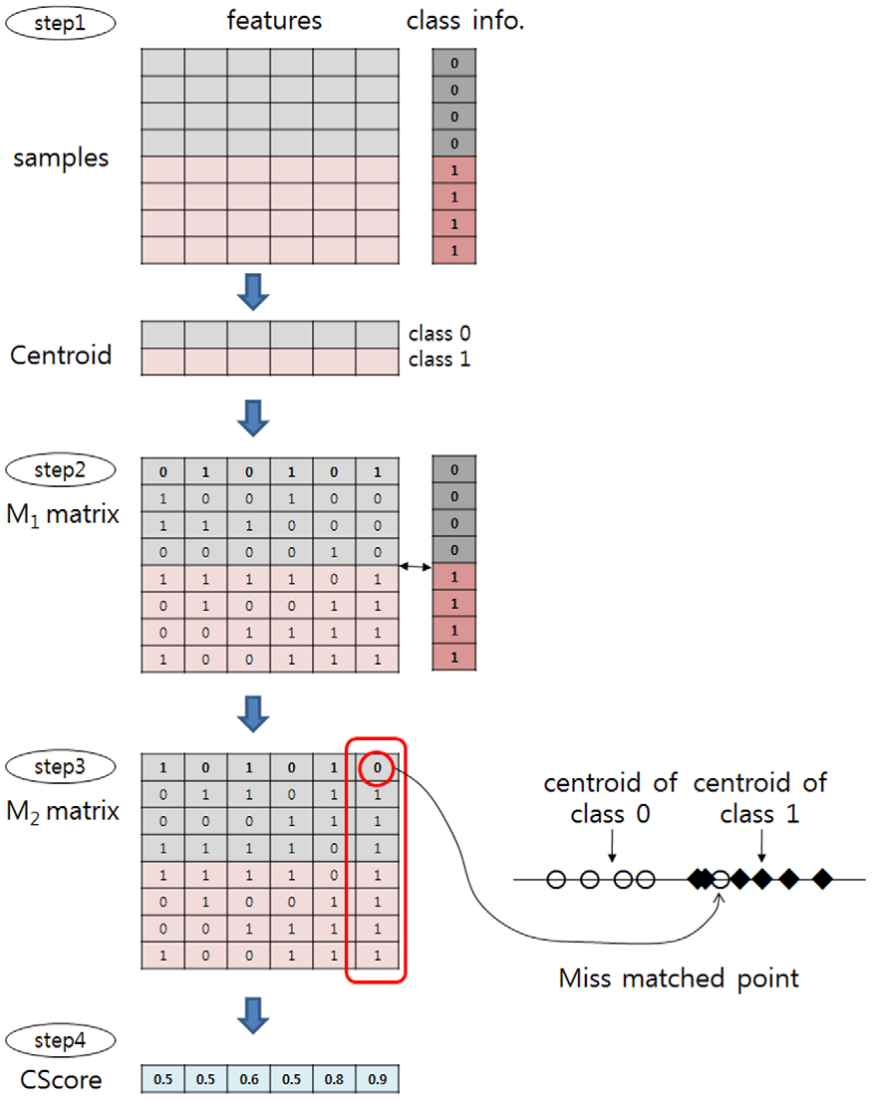
Procedure to get CScore().

Fig. 3 presents step 1 to step 4. The range of CScore(*f_i_*) is [0, 1]. If CScore(*f_i_*) is close to 1, this shows that classes in feature *f_i_* are clustered well and elements in *f_i_* can be clearly classified. Therefore, we can use CScore(*f_i_*) as a criteria to select features for classification work. CBFS chooses highly scored features using the CScore() function. Implementable algorithm to get CScore() is available in the Supplementary Material link (http://biosw.dankook.ac.kr/cbfs). CBFS can be combined to other feature scoring schemes. To distinguish combined algorithms as shown in the next section, we denote a pure CBFS algorithm as CBFS_org_.

### Improvement of CBFS with R-value

**Figure 4:**
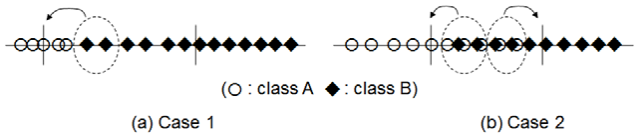
Two cases that CScore() produces wrong scores. If data range of a class is so smaller than neighbor class', CScore may produce wrong score (Case 1). If two classes' have wide overlapping area, CScore may produce wrong score (Case 2).

Though CBFS itself shows high performance for feature selection, we can improve its quality by combining other scoring schemes. In this section, we describe a combining example between CBFS and *R-value*. We can apply this approach to combine other scoring schemes. Scoring function of CBFS is based on distance between each data point and centroid of classes. In some cases, this produces the wrong scores, as shown in Fig. 4. In Fig. 4(a), class A and class B are clearly separated but two points of class B in the dotted circle are classified as class A and this decreases the value of the CScore(). If two classes are widely overlapped as shown in Fig. 4(b), many points in the overlapping area will be mis-classified. In the cases shown in Fig. 4, the *R-value* is a better scoring function because the *R-value* does not consider the distance to the centroid of classes but instead, to the number of nearest neighbors.

Traditional approaches to combine different feature selection methods usually just use intersection. Next box presents the simple steps required to combine CBFS and *R-value*. We denote this approach as CBFS_intersection_.

- Step 1. Choose *n* features using which have higher scores by CScore().
- Step 2. Choose *n* features using which have lower scores by *R-value.*
- Step 3. Extract *m* features from intersection of step 1 and step 2.

A difficulty in CBFS_intersection_ is how to determine *n* if we fix *m*. For example, even if we want to get 20 features using CBFS_intersection_, we do not know the correct number of *n* because we cannot estimate the number of intersections between step 1 and step 2. Therefore, we modify CBFS_intersection_ to extract the exact number of *m* features. We denote it as CBFS_exact_. Basic steps for CBFS_exact_ are as follows:

- Step 1. Initialize *n* as *m* and *ExtractList* as empty.
- Step 2. Choose *n* features using which have higher scores by CScore().
- Step 3. Choose *n* features using which have lower scores by *R-value.*
- Step 4. Extract features from intersection of step 2 and step 3, and if they are not in *ExtractList*, store them to *ExtractList.*
- Step 5. If number of element in *ExtractList* < *m*, then

*n* ← *n*+1

go to step 2

A confusing point in feature selection is to select better features from multiple features that have same ranking scores. Intersection with other feature selections offers a solution for the problem. Pseudo codes for CBFS_intersection_, CBFS_exact_, and *R-value* are available in the Supplementary Material link (http://biosw.dankook.ac.kr/cbfs). In the next section, we present benchmarking results for CBFS_org_, CBFS_intersection_, and CBFS_exact_.

### Datasets, feature selection algorithms, and classifiers

To compare feature selection algorithms we choose various kinds of datasets, which contain varying numbers of features and samples. Duke, Leukemia, DLBCL, and Carcinoma are well known microarray datasets. Other datasets come from the UCI repository [10] and several websites. Table 1 summarizes the benchmark datasets. FeaLect, FSDD, and Relief feature selection algorithms are compared with proposed CBFS_org_, CBFS_intersection_, and CBFS_exact_. FeaLect is widely considered as a state-of–the-art algorithm and details are described in Section 1. For simplicity we denote FeaLect as Lect from here on.

**Table 1:** Summary of the benchmark datasets.

No	Dataset	#of features	#of classes	# of samples	Reference
1	Arcene	10000	2	100	[17]
2	Prostate	12600	2	102	[18]
3	Madelon	500	2	2000	[17]
4	Duke	7129	2	88	[19]
5	Leukemia	7129	2	144	[20]
6	Sonar	60	2	416	[21]
7	DLBCL	661	3	282	[22]
8	Carcinoma	7457	2	72	[23]

The basis of the FSDD algorithm is to identify the features that result in good class separability among classes and to make the samples in the same classes as close as possible. A criterion used for selection of good features is d_b_ – β d_w_ and the criteria function can be expressed as follows:
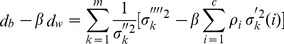
(5)where *m* is the number of selected features, *c* is the number of classes, and 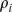 is the prior probability of the *i*th class.

Relief is regarded as one of the successful features of selection algorithms. The basic idea of Relief is to iteratively estimate feature weights according to their ability to discriminate between neighboring instances. In each iteration, an instance *x* is selected randomly, and its nearest instance is found from the same class (NH), as well as different classes (NM). Finally, the weight value is updated by the equation:
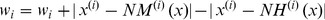
(6)


If *w_1_* < *w_2_*, *feature_2_* is better than *feature_1_*. The ReliefF (Relief-F) algorithm [11], which is an updated version of Relief, is more robust and can deal with incomplete and noisy data.

To compare classification accuracy between the current feature selection algorithms and proposed CBFS, we used the *k*-nearest neighbor (KNN) and support vector machine (SVM). In the KNN classification analysis, we used *k* = 5 for K because this value was found to produce the best accuracy in most of cases. For the SVM test, we use LIBSVM tool [12] with linear kernel. Whole user defined value is set as default such as degree, gamma, and coef0. We use the Lect algorithm that is imported in the R-package. User defined values of FSDD are *Beta*  = 3, and *K* = 3. In case of ReliefF, we use *K* = 7. Proposed CBFS_intersection_ chooses a threshold value *n* = 100. We use well-known validation methods, *k*-fold cross validation [13] where *k* = 5 to avoid the problem of over-fitting the classification.

## Results

**Figure 5:**
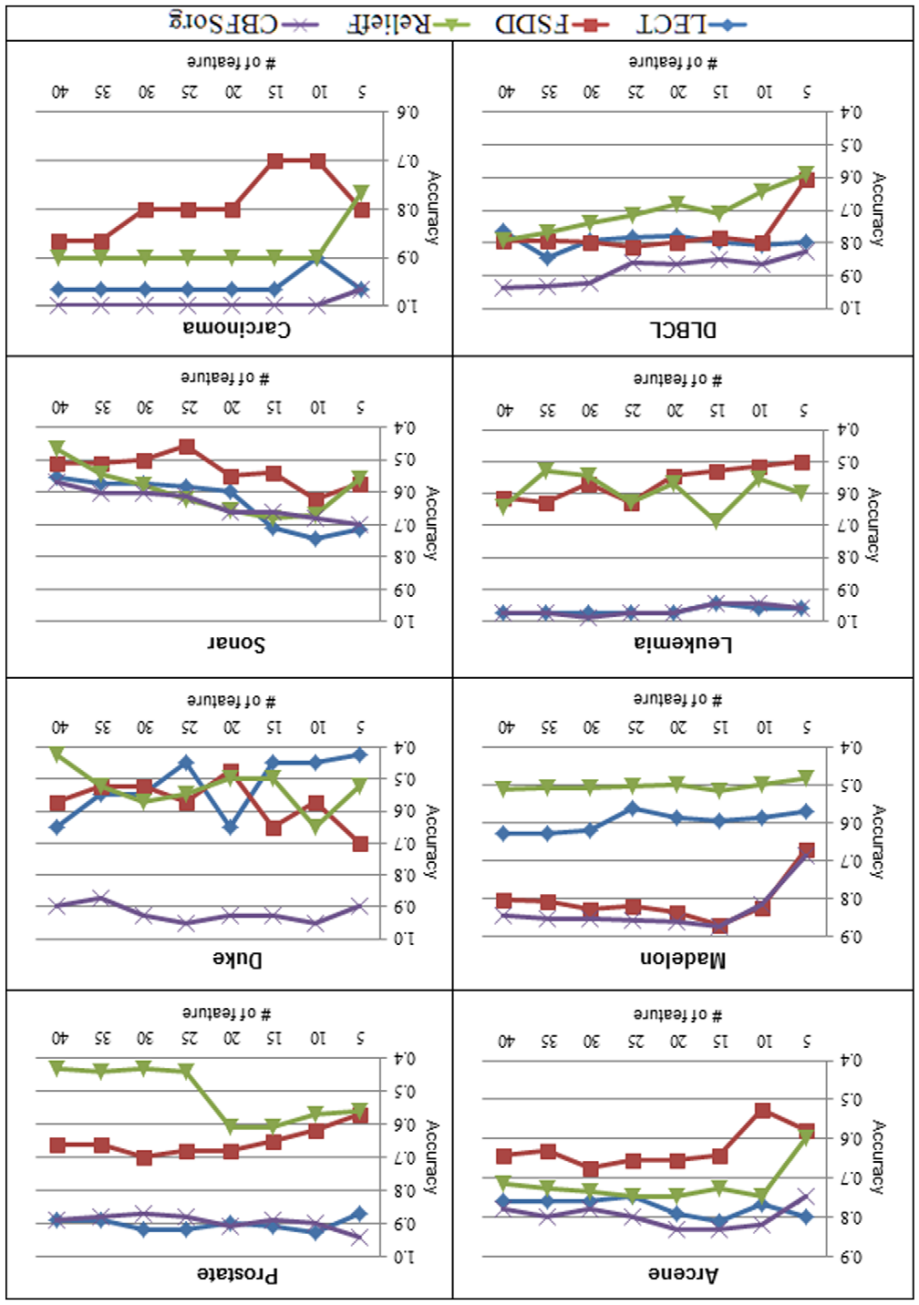
Comparison of classification accuracy using KNN where K = 5. In every case, CBFSorg shows best classification accuracy.

**Figure 6:**
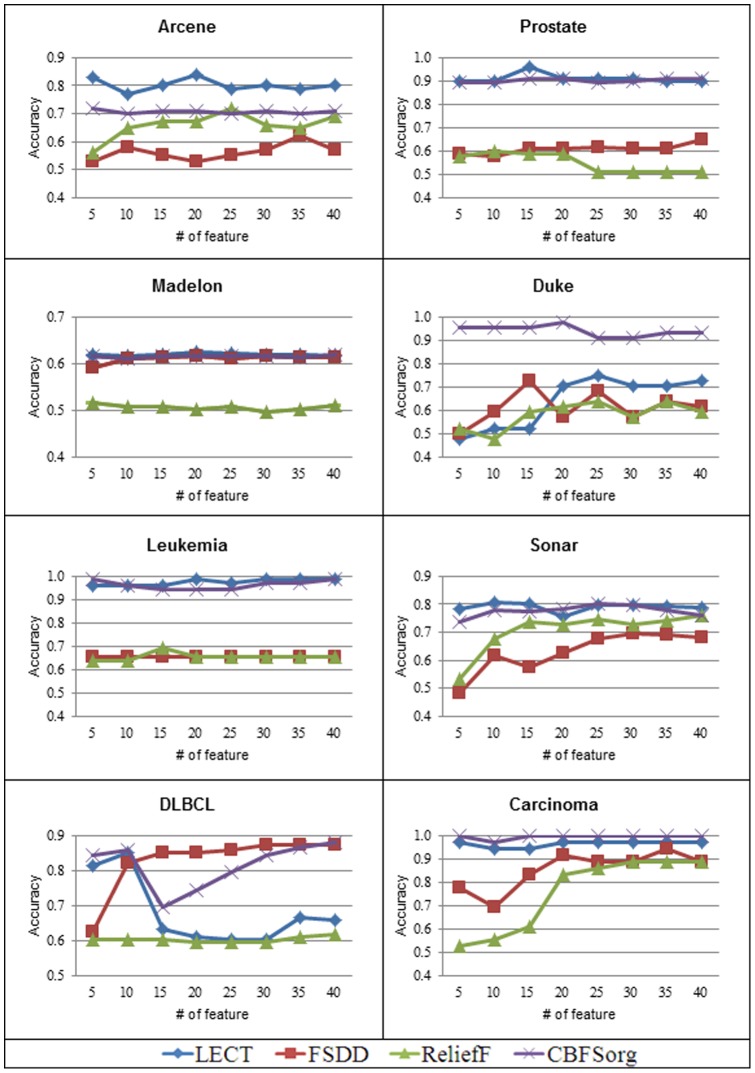
Comparison of classification accuracy using SVM with linear kernel. LECT and CBFSorg show similar performance, but CBFSorg is a little bit better than LECT.

Relevance, sparsity, and optimality are measures to evaluate feature selection algorithms. Relevance and sparsity are generally used for microarray area and requires domain knowledge. Optimality evaluates classification accuracy using the same number of features from different feature selection algorithms. Fig. 5 and 6 present the optimality evaluation. We test KNN and SVM to compare classification accuracy based on 5-fold validation. Fig. 5 and 6 show that the proposed CBFS_org_ is far better than the current filter methods such as FSDD and Relief. In addition, it also outperforms Lect, which is a superior quality feature selection method. Lect can be classified as a wrapper method. In general, wrapper methods produce better classification accuracy but require long execution time. Though the proposed method is a filter method, it exceeds or remains the performance of wrapper method. CBFS_org_ shows good classification accuracy both in KNN and SVM. CBFS_org_ has a generality for well-known classification algorithms.

**Figure 7:**
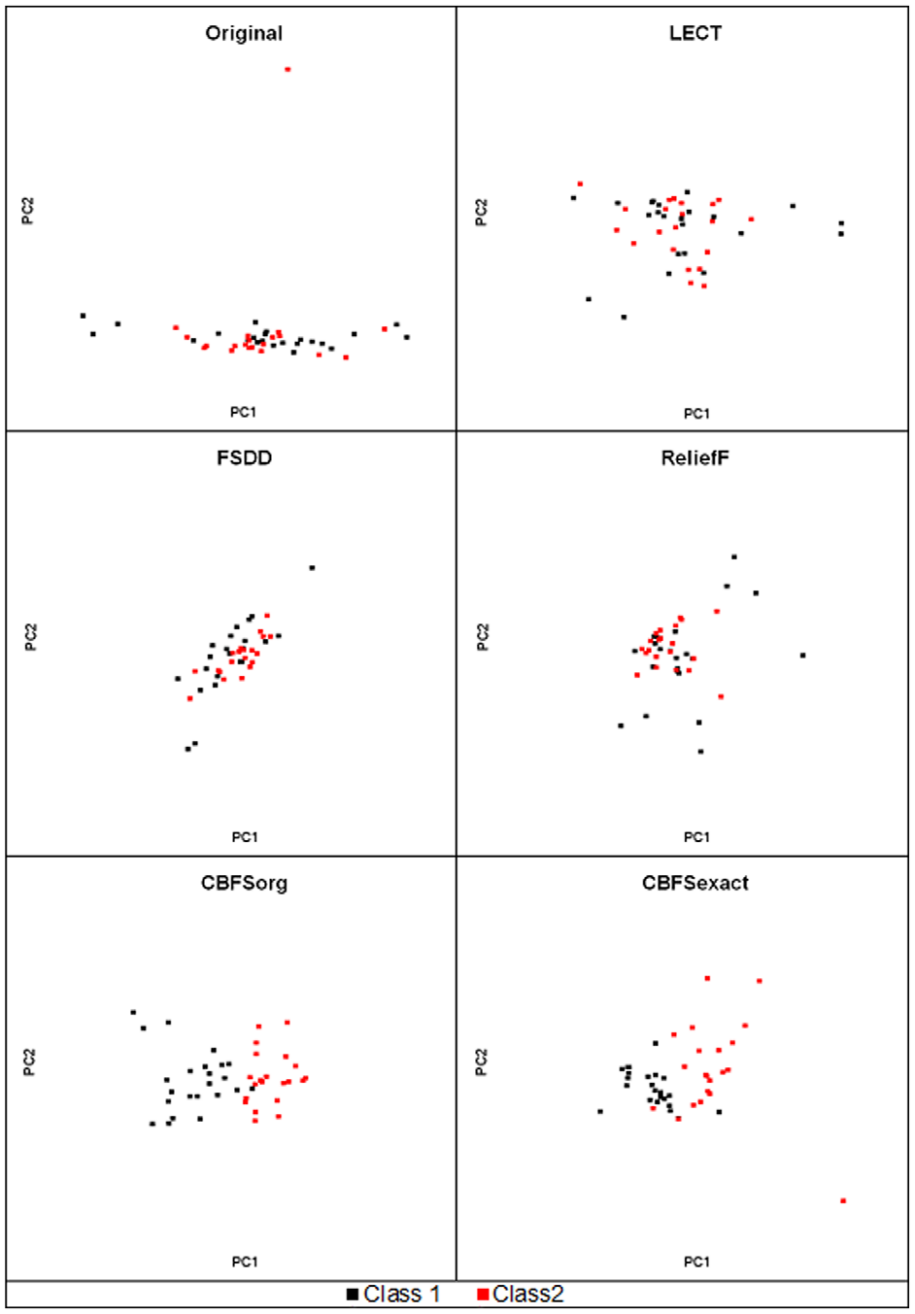
PCA analysis for feature selection results for the Arcene dataset. Congestion areas of red and black points are narrow in CBFS graphs comparing with the others.

Fig. 7 shows PCA analysis for feature selection results for the Arcene dataset. Red and black points represent samples of two different classes. Congestion areas of red and black points are narrow in CBFS graphs compared with the others. In general, the more narrow congestion area we get, the better classification accuracy we can expect. This is why the CBFS algorithm produces higher accuracy than other algorithms.

Table 2 summarizes the best classification accuracies by prepared feature selection algorithms on benchmark datasets. We test various parameter values and a number of features for each feature selection algorithm and classifiers, and choose the best accuracies. The proposed CBFS_org_ and CBFS_exact_ occupy top accuracies for each datasets except Prostate. In particular, CBFS_exact_ produces 22.7% and 23.8% higher than Lect on the Duke and Madelon dataset, respectively. It is clear that the number of features to produce best accuracy of CBFS_org_ and CBFS_exact_ are generally smaller than other algorithms. For example, Lect, CBFS_org_ and CBFS_exact_ produce best accuracy on the Leukemia dataset, and Lect uses 25 features whereas CBFS_exact_ uses only 5 features.

**Table 2:** Best classification accuracies and number of features to produce accuracies.

		Lect	FSDD	ReliefF	CBFS_org_	CBFS_intersection_	CBFS_exact_
**Arcene**	KNN	0.863(20)	0.758(20)	0.789(35)	0.832(15)	0.832(20)	**0.905(25)**
	SVM	0.840(20)	0.620(35)	0.720(25)	0.720(5)	0.700(5)	0.720(5)
**Prostate**	KNN	0.930(15)	0.750(30)	0.650(15)	0.940(5)	0.950(5)	0.940(15)
	SVM	**0.961(15)**	0.647(40)	0.598(10)	0.912(15)	0.922(10)	0.931(10)
**Madelon**	KNN	0.646(35)	0.874(15)	0.528(5)	**0.884(15)**	0.865(15)	0.865(20)
	SVM	0.624(20)	0.617(20)	0.519(5)	0.619(40)	0.612(20)	0.619(35)
**Duke**	KNN	0.725(20)	0.700(5)	0.675(25)	0.975(20)	0.925(5)	0.950(10)
	SVM	0.750(25)	0.727(15)	0.636(25)	**0.977(20)**	0.955(35)	0.955(10)
**Leukemia**	KNN	0.986(25)	0.643(40)	0.686(15)	0.986(30)	0.971(25)	**0.986(5)**
	SVM	0.986(20)	0.653(5)	0.694(15)	**0.986(5)**	0.972(30)	0.972(40)
**Sonar**	KNN	0.776(10)	0.673(10)	0.712(15)	0.737(5)	0.751(10)	0.732(5)
	SVM	0.807(10)	0.697(30)	0.760(40)	0.803(25)	0.808(15)	**0.813(20)**
**DLBCL**	KNN	0.859(10)	0.844(10)	0.815(40)	**0.941(40)**	0.867(15)	0.904(25)
	SVM	0.851(10)	0.872(30)	0.617(40)	0.879(40)	0.716(20)	0.851(5)
**Carcinoma**	KNN	1.000(25)	0.900(40)	0.933(40)	**1.000(5)**	1.000(20)	0.967(5)
	SVM	0.972(5)	0.944(35)	0.889(30)	**1.000(5)**	1.000(20)	0.972(5)

Table 2 also shows that CBFS_exact_ produces better accuracy than CBFS_org_ in some cases. CBFS_intersection_ has lower accuracy than CBFS_org_ and CBFS_exact_. We can consider combining multiple feature selection algorithms to improve classification accuracy.

**Figure 8:**
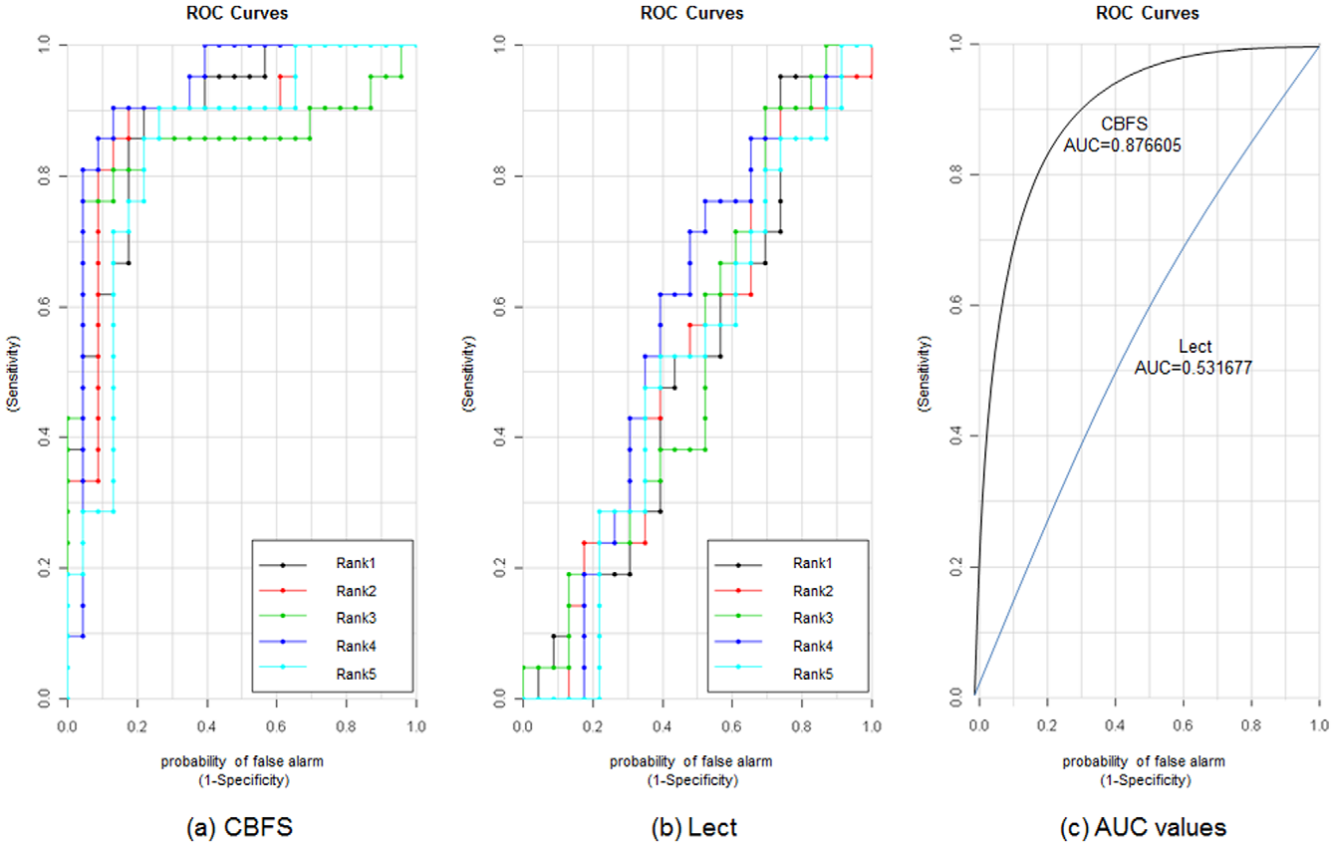
ROC curve and AUC values for CBFS and Lect feature selection algorithms on the Duke dataset.

**Figure 9:**
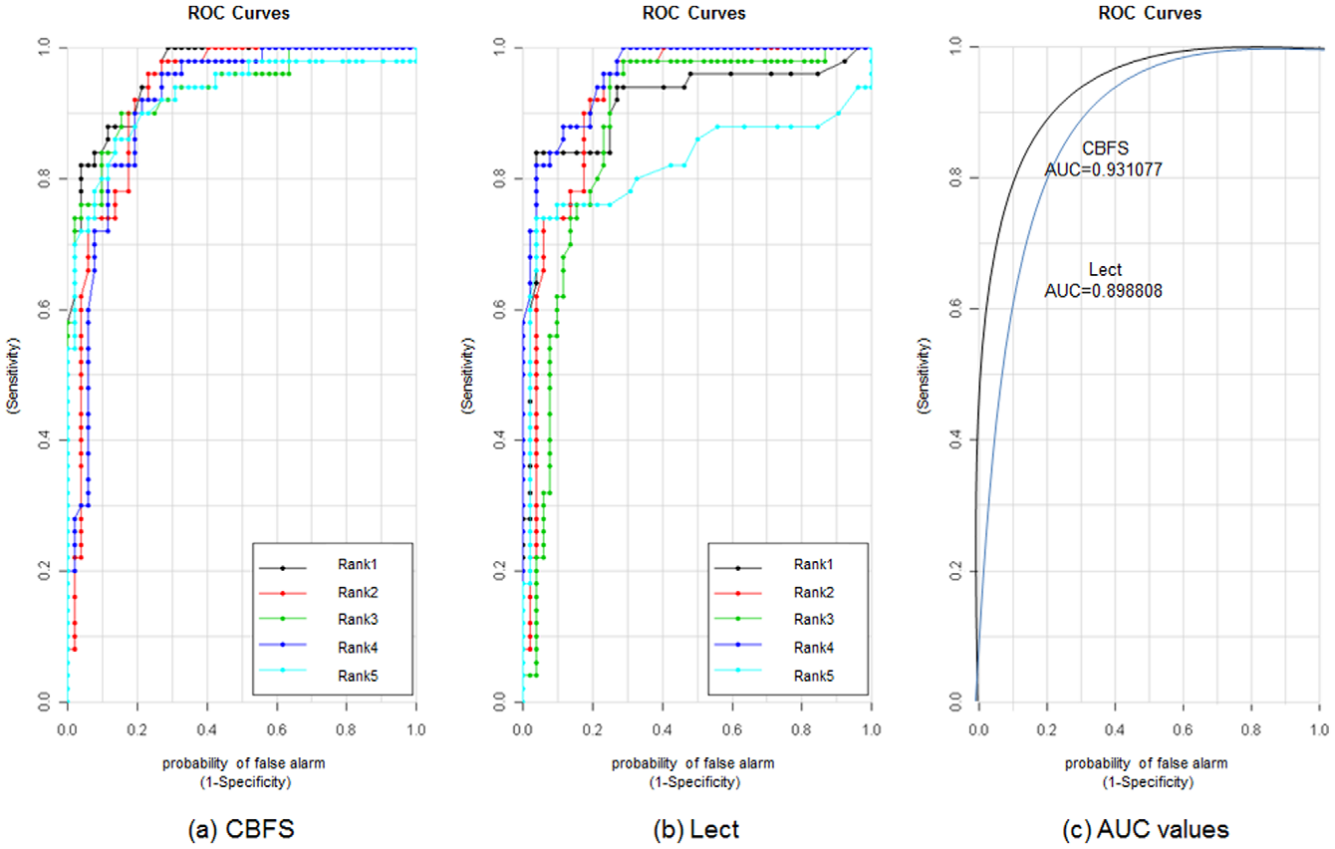
ROC curve and AUC values for CBFS and Lect feature selection algorithms on Prostate dataset.

Some microarray datasets have a small number of samples. For example, Carcinoma has only 72 samples. In that case, classification accuracy is not a reliable measure to evaluate feature selection algorithms, and instead we need to analyze the risk of mis-classification or prediction using the ‘loss function’ [14] or Receiver Operating Characteristic (ROC) curve [15]. ROC curves show a two-dimensional graph using sensitivity and 1- specificity. They are widely used in biology and medical science for evaluating prediction methods or markers. We used ROC curves to compare the stability of CBFS with that of Lect. Currently, Lect is a top ranked feature selection algorithm, and we only use it for comparison purposes. Fig. 8 and 9 show ROC analysis for CBFS and Lect on the Duke and Prostate dataset, respectively. We extract five features using CBFS and Lect, and list the relationship values between samples in the features and their class labels. We also draw AUC curves according to [15] which use the average values obtained from the ROC curves. Fig. 8(c) and Fig. 9(c) shows AUC value of CBFS is greater than Lect, which means that CBFS is a more stable and superior method than Lect.

## Discussion

### Time complexity of calculating CScore

CBFS is a fast and efficient algorithm. From the steps to calculate CScore() in section 2.1, we analyze time complexity. Let *n*, *c*, and *f* equal the number of samples, classes, and features of given dataset, respectively. The time complexity of each step for CBFS method is as follows:

1. Calculate centroid of each class in each feature: O(*m*•*n*)
2. Produce *n* × *m* matrix M_1_ and M_2_: O(*m*•*n*•*c*)
3. Calculate CScore() for each features: O(*m*•*n*)

Therefore, the total time complexity is *O*((2+*c*)•*m*•*n*). Table 3 shows computation times of feature selection for selected algorithms. CBFS is the fastest feature selection algorithm.

**Table 3:** Computation time of feature selection for the Madelon dataset.

(Unit: *ms*)	
FS algorithm	Computation time
LECT	1,732,000
FSDD	266
ReliefF	46,047
CBFS	141

Analyses were conducted using a computer with an Intel® Core™2 Duo CPU E7400 2.80 GHz, 3.46 GB RAM, and windows XP professional version 2002 service pack 3.

### Overfitting problem of proposed algorithm

Overfitting is a general problem of machine learning algorithms such as classification. To avoid overfitting, K-fold validation and LOOCV skims are used in classification tests. Validation errors can be used to evaluate feature selection algorithms. Table 4 shows the classification accuracy and validation errors of Lect and CBFS on benchmark datasets. We calculate validation errors from five to twenty features derived by Lect and CBFS. Lect uses the L1-regularization technique to avoid overfitting problem, so we can indirectly evaluate the validation error of CBFS by comparing Lect and CBFS. CBFS gives lower validation errors than Lect for every dataset except Sonar. The average validation error of Lect and CBFS are ±8.33% and ±6.85%, respectively. If a feature selection algorithm has a lower validation error, it means that the algorithm is less sensitive for distribution of samples and may produce less overfitting problems. Most distance-based filters assume that if a feature has short intra-classes and long inter-classes distances, it can produce high classification accuracy, but this assumption carries the risk of higher overfitting. Proposed CScore() evaluates each feature based on the degree of condensation of samples to the centroid of classes, and reduces validation error.

**Table 4:** Classification accuracies and validation errors for Lect and CBFS.

	Lect	CBFS
Arcene	77.4±8.8%	79.9±8.3%
Prostate	77.3±8.8%	79.8±8.3%
Madelon	59.6±1.6%	83.0±1.6%
Duke	52.1±.5%	91.8±6.5%
Leukemia	96.4±3.6%	96.4±3.5%
Sonar	63.3±14.4%	63.8±18.4%
DLBCL	79.6±8.8%	88.4±7.8%
Carcinoma	95.8±4.1%	99.5±0.4%

### Application of CBFS

CBFS can be applied to any areas of data analysis that require feature selection scheme such as microarray gene selection, text categorization, and image classification. Microarray data is used to screen thousands of genes and determine whether genes have relationship with specific disease such as cancer. A gene corresponds to a feature and CBFS may suggest candidate genes according to feature evaluation values. Medical expert will analyze the biological functions of the candidate genes and find target genes that are related with diseases. Feature selection is an essential part of text classification. Document collections have 10,000 to 100,000 or more unique words. Many words are not useful for classification. Restricting the set of words that are used for classification makes classification more efficient and can improve generalization error [16]. Image retrieval is one of application area of CBFS. In image retrieval, each image data may have so many features to characterize the data. In feature extraction step, we don't know which features are efficient to characterize each image. After applying CBFS, we can evaluate quality of each feature and select best features for image retrieval system.

CBFS java program is available at http://biosw.dankook.ac.kr/cbfs
